# Dehydrogenases in the Flavoprotein Amine Oxidoreductase Superfamily

**DOI:** 10.1021/acs.biochem.5c00129

**Published:** 2025-06-12

**Authors:** Javeria Akram, Tavishi Budagavi, Zhiyao Zhang, Morgan Fowler, Andrew J. Gaunt, Todd J. Barkman, Frederick Stull

**Affiliations:** † Department of Chemistry, 4175 Western Michigan University, Kalamazoo, Michigan 49008, United States; ‡ Department of Biological Sciences, Western Michigan University, Kalamazoo, Michigan 49008, United States

## Abstract

Enzymes of the ubiquitous flavoprotein amine oxidoreductase (FAO) superfamily catalyze C–N bond oxidation of amine-containing substrates using flavin adenine dinucleotide (FAD) as a prosthetic group. Their reaction proceeds via a two-step mechanism involving hydride transfer from the substrate to the bound FAD cofactor, and the reduced flavin is subsequently reoxidized by a physiological electron acceptor. For nearly a century, it has been generally accepted that all enzymes in the FAO superfamily are oxidases, i.e., donating the electrons from substrate oxidation to dioxygen (O_2_). However, we recently showed that the FAO family enzymes nicotine oxidoreductase and pseudooxynicotine amine oxidase are not oxidases because they do not utilize oxygen efficiently, and instead are cytochrome *c* utilizing dehydrogenases. Here we characterized other bacterial FAOs and show that many are actually dehydrogenases that react poorly with O_2_ in favor of using a cytochrome *c* (CytC) protein closely encoded in the genome, indicating that there are many exceptions to the “oxidase” paradigm that has been traditionally ascribed to the FAO superfamily. These dehydrogenases are highly specific for the CytC from the same organism and are phylogenetically clustered with other FAOs that appear to be oxidases. Our findings further undermine the long-held view that all FAO family enzymes are oxidases and suggest that evolutionary switches between different oxidants are surprisingly frequent in this enzyme family.

## Graphical abstract

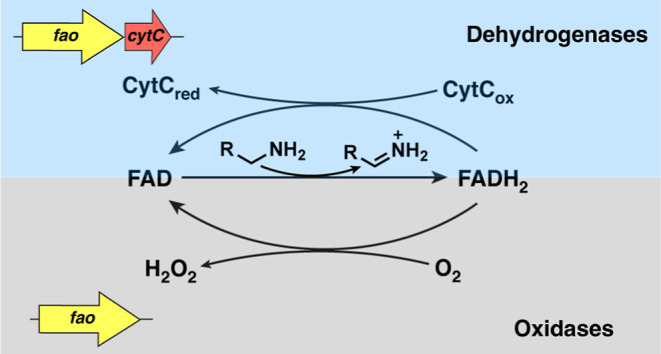

## Introduction

Flavoprotein amine oxidoreductases (FAOs) comprise a superfamily of enzymes that facilitate a diverse range of biologically significant chemical transformations in all kingdoms of life.^1–3^ FAOs use a riboflavin derived flavin adenine dinucleotide (FAD) prosthetic group as an electron shuttle to carry out the oxidation of a carbon–nitrogen bond in their amine-containing substrates, including human mitochondrial monoamine oxidase (MAO), which was first discovered in 1928.^4^ Since then, considerable research has been conducted on the mechanistic details of these enzymes, which are thought to be well understood.^5,6^ The FAO catalytic cycle can be divided into reductive and oxidative half-reactions with respect to the flavin cofactor. The FAD cofactor receives electrons from oxidizing the amine substrate in the reductive half-reaction, thereby forming reduced FAD hydroquinone (FADH_2_). Subsequently, these electrons are transferred to a physiological electron acceptor in the oxidative half-reaction, resulting in the regeneration of FAD to its original oxidized state. Enzymes within the FAO family typically exhibit oxidase activity, rapidly and efficiently utilizing O_2_ as the physiological electron acceptor for their FADH_2_ to form H_2_O_2_, hence the name “oxidases”.^2,3^ However, we recently discovered two FAO enzymes that challenge the general understanding of this superfamily: nicotine oxidoreductase (NicA2)^7–9^ and pseudooxynicotine amine oxidase (Pnao),^10^ which are both from a nicotine catabolizing pathway in S16.^11^ NicA2 and Pnao both react very poorly with O_2_. Instead, we discovered that the physiological electron acceptor is a cytochrome *c* (CytC) protein, meaning that NicA2 and Pnao are actually dehydrogenases despite being members of the FAO superfamily. NicA2 and Pnao both feature an N-terminal signal peptide characteristic of the twin arginine translocation (TAT) pathway^12^ while CytC, encoded by *cycN*, is sandwiched in between *nicA2* and *pnao* in the genome of S16 and has a signal sequence associated with the general secretory (sec) pathway.^13^ Thus, NicA2, Pnao and CycN are all predicted to be exported into the periplasm of S16, which presumably allows CycN to transfer electrons from substrate oxidation by NicA2 and Pnao into the electron transport chain (ETC) for additional ATP production.

We therefore wondered if NicA2 and Pnao are isolated outliers in the FAO superfamily, or if there are many other FAO enzymes that are, in fact, dehydrogenases. Using the two genetic criteria (I) presence of TAT signal peptide and (II) associated CytC gene with a sec signal peptide as indicators of potential dehydrogenases, we conducted a bioinformatic analysis of NicA2 related FAO sequences present in the NCBI database. This analysis, combined with biochemical characterization of 25 FAO proteins via stopped-flow experiments, reveals that there are many enzymes in this family that possess CytC-using dehydrogenase activity as opposed to oxidase activity. We further show that the dehydrogenases exhibit a high degree of preference toward the associated genome-encoded CytC and that in spite of the potential energetic benefits of CytC utilization, dehydrogenase activity has been evolutionarily lost many times in favor of O_2_ utilization.

## Materials and Methods

### Bioinformatic and Phylogenetic Analyses of FAO Sequences

The previously characterized dehydrogenase, NicA2, from S16 sequence was used as a query to obtain the top 5000 hits in the NCBI BLAST nonredundant database. Associated cytochrome *c* genes were identified from the genome annotations in the NCBI genome database by manually looking at the gene annotations in GenBank immediately upstream and downstream of the gene for the FAO in a given organism. In some cases, the open reading frame for CytC was not annotated in the genome in GenBank and was manually identified from within the unannotated sequence immediately upstream and downstream of the FAO locus. In these cases, a BLAST search was performed to confirm that the unannotated sequence indeed corresponded to a CytC. The TAT and Sec pathway signal peptides for FAO and CytC sequences were identified using the DTU Health Tech SignalP 6.0 software.^14^ Jalview was used to remove duplicate sequences using >98% sequence identity as the cutoff for identifying duplicate sequences.^15^ The remaining 2911 nonduplicate sequences were aligned with MAFFT,^16^ and the phylogenetic tree was generated using IQTree^17^ version 2.1.2 using the optimal substitution model (LG + F + I + G4) identified by ModelFinder in IQTree via the CIPRES Science Gateway.^18^ Branch support values were estimated using 1000 ultrafast bootstraps. To assess statistical significance for alternative topologies, the approximately unbiased test^19^ was used as implemented in IQTree. We used constraint trees to assess; (1) whether all putative dehydrogenases were monophyletic and also (2) whether only the dehydrogenases encoded in operons with c-type CytC were monophyletic (clades 2 and 3). To estimate ancestral states shown in Figures 1 and 3, we used maximum likelihood^20^ with multistate encoding as implemented in Mesquite.^21^

**Figure 1:**
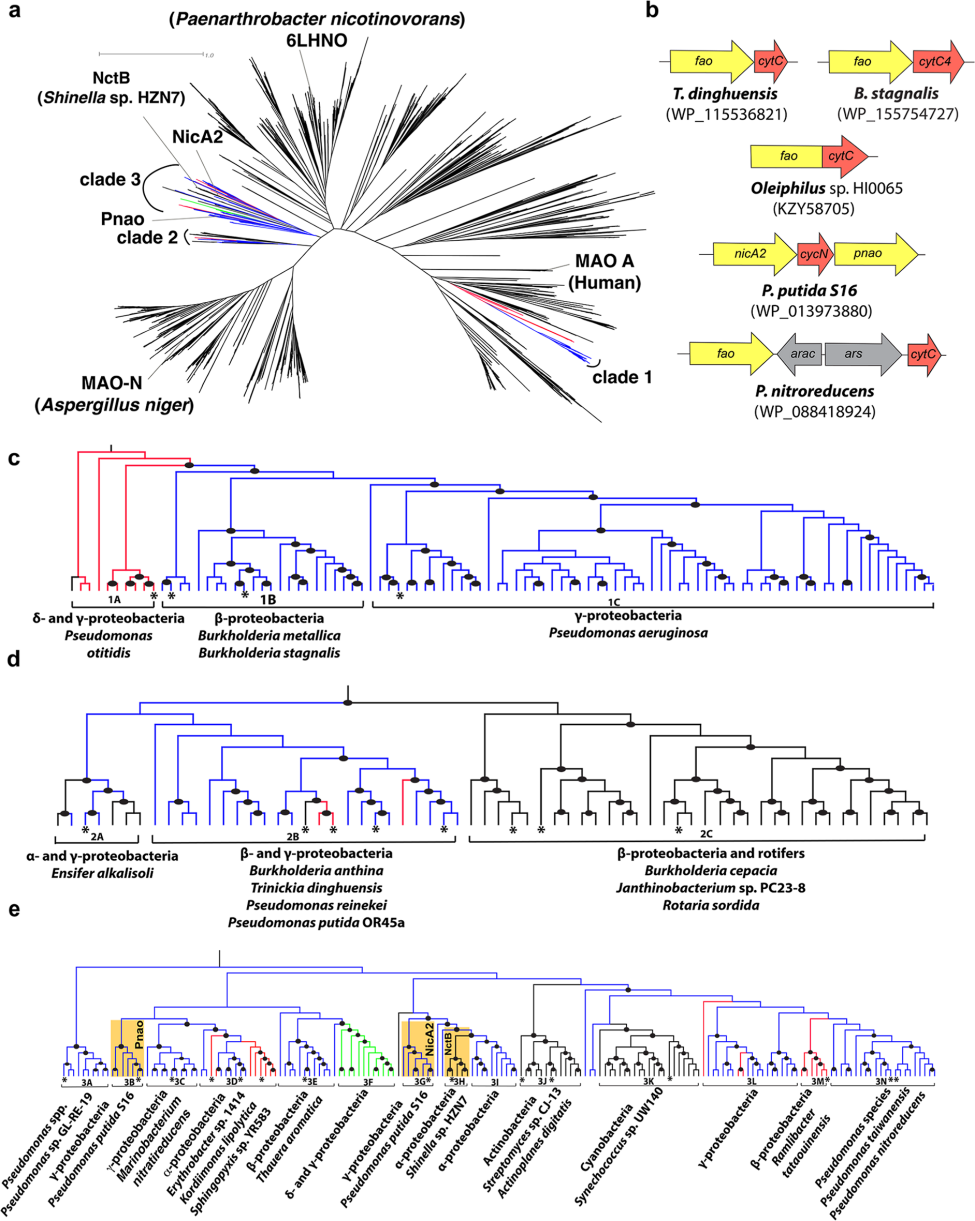
Phylogeny of FAOs. (a) Radial phylogram of 2911 FAO sequences showing clades 1–3 that are predicted to include dehydrogenases. (b) Operon structure of exemplar FAO-CytC pairs. (c), (d), and (e) clade 1, 2, and 3, respectively, with subclade labels shown and branches colored according to inferred ancestral operon configurations. Sequences that were functionally characterized in this study, and NicA2 and Pnao, are marked with an asterisk. Black branches: FAO with no signal peptide and no adjacent CytC. Blue branches: FAO with TAT signal peptide and adjacent CytC (putative dehydrogenases). Green branches: FAO with TAT signal peptide and CytC fused together as a single polypeptide. Red branches: FAO with TAT signal peptide but no adjacent CytC. Filled circles indicate nodes with significant (>0.9) branch support. Previously functionally characterized sequences NicA2,^7^ Pnao^10^ and NctB^27^ are highlighted in yellow. See also Figures S1–S3.

**Figure 2:**
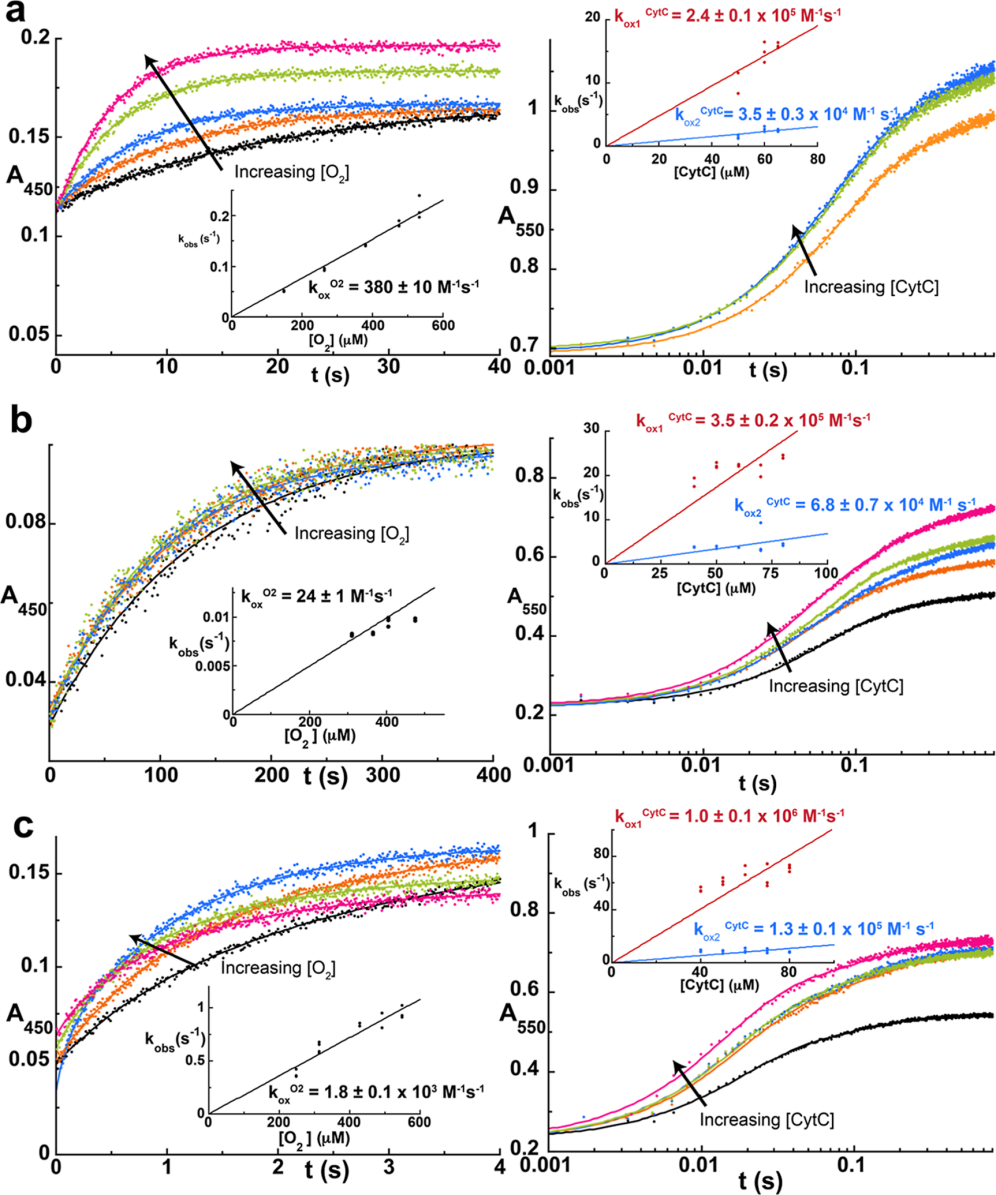
Kinetics of reoxidation for representative putative dehydrogenases by O_2_ and associated CytC. Overlay of reoxidation reaction traces of flavin hydroquinone for exemplar putative dehydrogenases from (a) , (b) *Pseudomonas* sp. GL-RE-19 and (c) . Left, traces for the reaction with O_2_ monitored at 450 nm and right, traces for the reaction with associated CytC monitored at 550 nm. The insets show the dependence of the observed rate constant(s) on oxidant concentration and linear fitting provides the bimolecular rate constant for the reaction with a given oxidant (*k*_ox_^O_2_^, *k*_ox1_^CytC^ and *k*_ox2_^CytC^). CytC traces were adjusted such that all concentrations begin at the same absorbance value to facilitate comparison. Note the logarithmic time scale for traces with CytC. See also Figure S4 for data on additional putative dehydrogenases.

**Figure 3:**
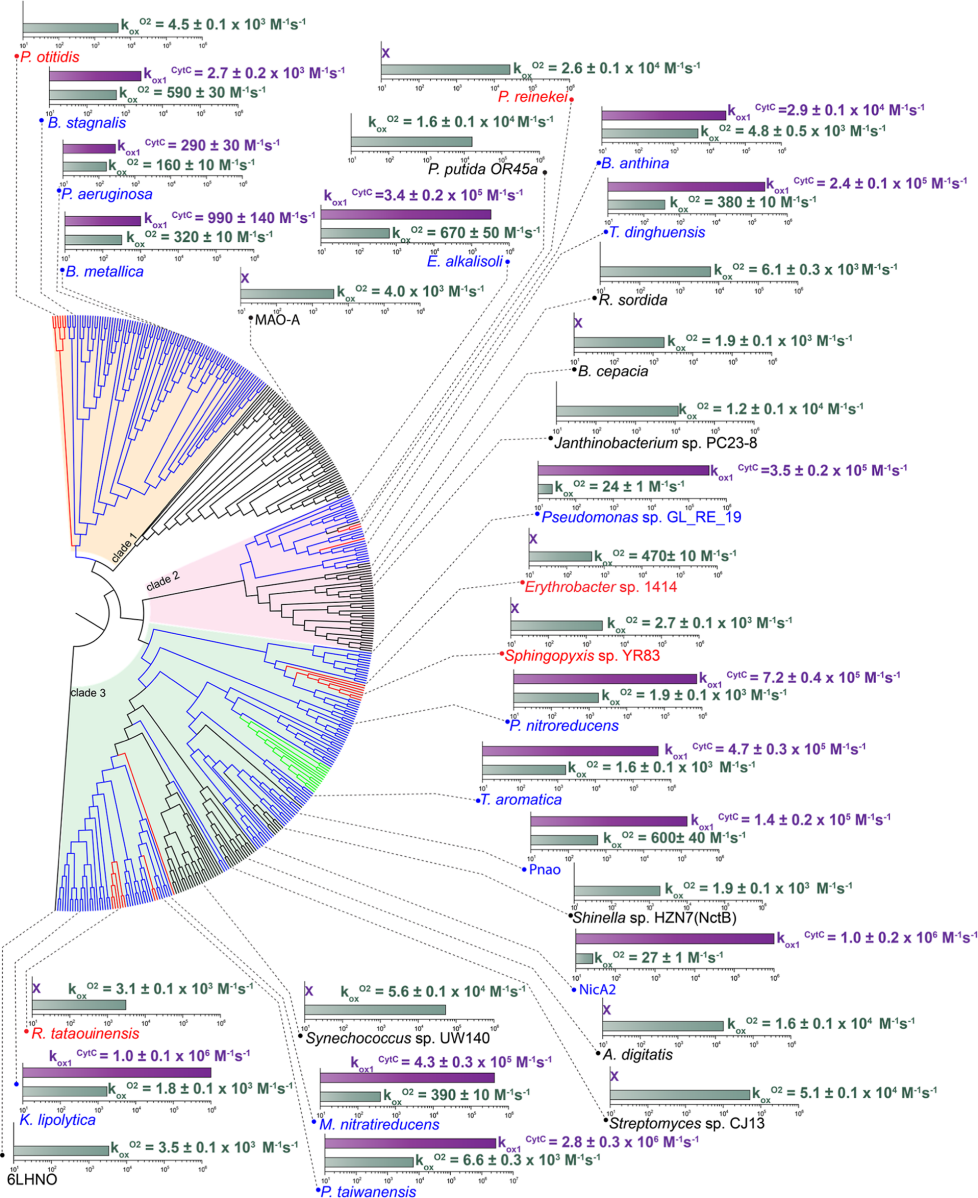
Comparison of rate constants measured for putative dehydrogenases and oxidases. Radial cladogram of clades 1, 2, and 3 (highlighted in tan, pink and sage) in addition to a clade that includes the bona fide oxidase, MAO-A and 6LHNO. Bar charts display the *k*_ox_^O_2_^ (greyish green) and *k*_ox1_^CytC^ (purple) for representative putative dehydrogenases and oxidases characterized in this study as well as previously studied NicA2,^7^ Pnao,^10^ and MAO-A.^30^ A purple X indicates that enzyme assays with CytC were not conducted because the corresponding FAO lacks an associated CytC in its operon. All bimolecular rate constants are shown for the enzymes without the product bound.

### Protein Expression and Purification

The FAO and cytochrome *c* genes for biochemical characterization were commercially synthesized and cloned into the NdeI and XhoI sites of pET-29b by Twist Biosciences to generate constructs with C-terminal His tags using the His tag encoded by the vector. FAO constructs were synthesized such that they included the predicted N-terminal TAT signal peptide in the final construct. Cytochrome *c* genes were ordered such that their predicted N-terminal Sec signal peptides (identified using SignalP 6.0 ^14^) were removed and replaced with the PelB signal peptide of (MKYLLPTAAAGLLLLAAQPAMA) to facilitate CytC transport to the periplasm as a requirement for heme loading. FAO expression constructs were transformed into BL21 (DE3) cells for protein expression. CytC expression constructs were cotransformed with pEC86 helper plasmid into BL21 (DE3) to promote the proper folding and biogenesis of CytC proteins.^7^ 50 mg/L kanamycin was used as antibiotic selection for pET-29b constructs and 34 mg/L chloramphenicol was also used when pEC86 helper plasmid was included. cells were grown in 6 L of protein expression media (12 g/L tryptone, 24 g/L yeast extract, 40 mL/L glycerol, 12.54 g/L K_2_HPO_4_, and 2.13 g/L KH_2_PO_4_) by incubating at 37 °C in a shaking incubator until optical density at A_600_ reached 0.6–0.8. The temperature was then lowered to 20 °C in case of FAOs and 30 °C in case CytC and induced with 100 μM and 10 μM IPTG, respectively. After overnight incubation, cell pellets were harvested by centrifugation at 5000 rpm and resuspended in lysis buffer (300 mM NaCl, 50 mM NaPO_4_, pH 8, 20 mM imidazole, 10% glycerol). The resuspended cells were then mixed with 100 μL of EDTA-free protease inhibitor cocktail (Abcam) and 1 μL of Benzonase nuclease (Sigma) and lysed by sonication for 5 min (10 s cycle with 30 s intervals). Cell lysate was then centrifuged at 18,000 rpm, and the resulting supernatant was loaded onto a pre-equilibrated (with lysis buffer) nickel affinity column. The loaded column was then washed with 10–15 column volumes of lysis buffer to eliminate the unwanted proteins. Bound FAOs and CytC were then eluted with lysis buffer containing 250 mM imidazole and subsequently concentrated to ∼3 mL volume using Amicon centrifugal concentrators. This step was followed by buffer exchange into the stopped-flow buffer (40 mM HEPES, 100 mM NaCl, pH 7.5, 10% glycerol). Purified FAO proteins were concentrated, flash frozen using liquid nitrogen and stored in −80 °C until required. CytC proteins were concentrated and stored at 4 °C. Prior to experimentation, CytC proteins had their hemes oxidized with potassium ferricyanide followed by buffer exchange to remove excess ferricyanide. The yields obtained from the above procedure were typically 1–5 mg of protein per liter of bacterial culture for CytC proteins and 1–10 mg of protein per liter of culture for FAO proteins. FAO concentrations were estimated using the extinction coefficient for FAD (ε_450_ 11,300 M^–1^ cm^–1^).^22^ CytC concentrations were estimated using ε_410_ 106,000 M^–1^ cm^–1^.^23^

### Transient State Kinetics

All stopped flow experiments were carried out using the TgK Scientific SF-61DX2 KinetAsyst stopped-flow instrument in stopped-flow buffer, at 4 °C. The absorbance changes were monitored using the instrument’s multiwavelength CCD detector. The identity of the amine-containing substrate is unknown for most of these FAOs and dithionite, which is commonly used as an artificial reductant for flavoenzymes, was unable to reduce flavin semiquinone to hydroquinone for many of them, similar to that observed previously with Pnao.^10^ However, pseudooxynicotine, the natural substrate for Pnao, was found to react slowly with most of the FAOs characterized and was therefore used as an artificial reductant to generate FADH_2_ in these FAOs through anaerobic titrations except for the FAOs from *Janthinobacterium* sp. PC23-8, , , and . These FAOs did not react with pseudooxynicotine but could be reduced fully to FADH_2_ by dithionite, and dithionite was used for stoichiometric flavin reduction for these enzymes. To reduce the flavin cofactor, 36 μM purified FAO in stopped-flow buffer was placed in a glass tonometer. The tonometer was then attached to the anaerobic Schlenk line to purge alternate cycles of vacuum and argon;^24^ this process eliminates the atmospheric and dissolved dioxygen which can otherwise act as an oxidant and influence the experimental observations. Artificial reductant dithionite (1 mg/mL) or pseudooxynicotine (10 mM, bubbled with argon for 10 min) in stopped-flow buffer was titrated from a gastight syringe into the FAO containing tonometer. The extent of reduction was monitored from the absorbance change at 450 nm via a spectrophotometer, and the FAO was titrated with reductant up to the point where the flavin was fully reduced to the hydroquinone form.

Reduced FAO containing tonometer was then loaded onto preblanked (with anaerobic stopped-flow buffer) stopped-flow instrument. In the case of dioxygen reactivity experiments, FAO was allowed to react with various concentrations of buffer dissolved dioxygen. O_2_ concentrations were prepared in a gastight syringe containing stopped-flow buffer by bubbling the buffer with various known O_2_/N_2_ ratios prepared using a gas blender. A Hansatech Oxygraph+ O_2_ electrode was used to measure the O_2_ concentration present in the buffer bubbled with each O_2_/N_2_ ratio prior to loading onto the stopped-flow instrument. The oxidation of the reduced flavin cofactor by O_2_ was monitored by following the increase in absorbance at 450 nm that accompanies flavin oxidation. In case of CytC reoxidation, FAOs were mixed with various concentrations of anaerobic oxidized CytC (typically ranging from 80 to 160 μM) prepared in a tonometer as described above. The reaction was monitored by measuring the change at 550 nm that accompanies heme reduction from the ferric to ferrous states.

### Data Analysis

Data obtained from stopped flow experiments was analyzed using KaleidaGraph. Traces for reactions with O_2_ fit to a single exponential function (eq 1) whereas traces for reactions with CytC generally fit to a sum of two exponentials (eq 2), similar to that observed previously for the reaction of NicA2 ^7^ or Pnao^10^ with CycN, likely due to two stepwise one-electron oxidations of FADH_2_ by two CytC molecules. The observed rate constant(s) (*k*_obs_) for reaction traces with O_2_ and CytC varied linearly with substrate concentration, indicating that the initial encounter between substrate and FAO is the rate limiting step for each flavin oxidation event. Traces involving reaction between FAO and CytC from different organisms were slow, only displaying a single exponential and were therefore fit to eq 1. In eqs 1 and 2, Δ*A* is the kinetic amplitude for each phase, *k*_obs_ is the apparent first order rate constant, and *A*_∞_ is the absorbance at the end of the reaction. The *k*_obs_ values obtained for each concentration were then plotted against O_2_/CytC concentration and a linear fit determined the *k*_ox_ for each FAO.

1 $$Y={\Delta Ae}^{-k_{obs}*t}{+A}_{\infty }$$

2 $$Y={\Delta A}_{1}e^{-k_{obs1}*t}+{\Delta A}_{2}e^{-k_{obs2}*t}{+A}_{\infty }$$

### AlphaFold Model Prediction

AlphaFold models were generated using AlphaFold 3 ^25^ using two copies of FAO sequence, one copy of CytC sequence along with two FAD and one heme ligand as input.

## Results

### Identification and Phylogeny of Putative Dehydrogenases in the FAO Superfamily

The known dehydrogenases NicA2 and Pnao both contain N-terminal TAT signal peptides and their genes form an operon with a gene encoding for CytC—sequence information that helped lead to their identification as dehydrogenases. We therefore searched for these same sequence indicators in other bacterial FAOs to identify other potential dehydrogenases. We first obtained the top 5000 sequences from a BLAST search using NicA2 as a query, which, after removing duplicates, resulted in 2911 nonredundant FAO sequences. Among these, we identified those containing an N-terminal TAT signal peptides using SignalP 6.0 ^14^ and detected the presence or absence of adjacent CytC gene by manually inspecting their genomes of origin. This bioinformatic analysis of 2911 FAO superfamily sequences from GenBank identified more than 100 FAO sequences that are predicted to have both a TAT signal peptide and adjacent CytC gene, thereby flagging them as potential CytC-using dehydrogenases. Phylogenetic analysis of these putative dehydrogenases shows that they are all of bacterial origin and spread across three clades that include NicA2 and Pnao and are distant from well-characterized FAOs such as human^5^ and fungal^26^ monoamine oxidases (MAOs) (shown in blue in Figures 1 and S1–S3). Statistical analyses^19^ strongly reject a single origin for all putative dehydrogenase-type FAOs (*p* < 0.05), thus there appear to be as many as three lineages: clade 1 as well as the phylogenetically distant clades 2 and 3 (Figure 1). We cannot reject the potential monophyly of dehydrogenase-containing clades 2 and 3, though the optimal tree shown in Figure 1 presents them as separate (*P* > 0.05). In most cases, the CytC gene was found encoded adjacent to the FAO gene, forming an operon in the genome of origin, likely indicating coexpression and thus possible functional interaction of the two proteins. While Figure 1 shows that there are at least two independent clades that contain predicted dehydrogenases, these clades also include other members that lack both TAT signal peptides and adjacent CytC genes (shown in black in Figures 1 and S1–S3), leading us to hypothesize that they are more recently evolved O_2_-using oxidases. In addition, there are a small number of sequences that contain only the TAT signal peptide with no detectable CytC gene in the vicinity of the FAO suggesting they may be periplasm-localized oxidases or may use CytC encoded elsewhere in the genome (shown in red in Figures 1 and S1–S3). Interestingly, as further evidence of a functional relationship between the encoded FAO and CytC, subclade 3F within clade 3 (shown in green in Figure 1e) has the two gene sequences directly fused and are thus predicted to be expressed as a single polypeptide, potentially allowing for direct electron transfer from FADH_2_ in the FAO domain to heme in the CytC domain.

### Kinetic Verification of Dehydrogenase Activities

Representatives from each putative dehydrogenase subclade were selected for experimental characterization. Each purified FAO containing reduced FADH_2_ (FAO-Fl_red_) was reacted with various concentrations of O_2_ or its associated CytC to measure the kinetics of FAO-Fl_red_ oxidation by these two substrates in the oxidative half-reaction using stopped-flow instrumentation. Putative dehydrogenases in clade 2 and 3 from , *Pseudomonas* sp. GL-RE-19, , , , , , and were rapidly oxidized by their respective genome-adjacent CytC, with reaction traces generally completing in under a second and occurring in two kinetic phases. We previously observed similar biphasic behavior with NicA2 and Pnao, which can be attributed to two stepwise one electron transfers from FADH_2_ to two molecules of CytC.^7,10^ The amplitudes of the traces titrated with the CytC concentration, reaching a plateau at the higher CytC concentrations where the first phase contributes ∼60% of the total signal change and the second contributes ∼40% of the signal change. Since the lowest CytC concentration used in these experiments was more than 2-fold greater than the enzyme concentration, the fact that the amplitudes titrate with CytC concentration likely indicates that electron transfer from reduced flavin to CytC is not an irreversible process; i.e., for the reactions with lower CytC/FAO ratios, an equilibrium is reached at the end of the reaction where some of the electrons still reside on the flavin of the FAO. The observed rate constant (*k*_obs_) for both kinetic phases increased linearly with CytC concentration, similar to that observed with NicA2 and Pnao, indicating that electron transfer in both steps is rate-limited by the initial binding of CytC to the FAO.^7,10^ That the kinetic amplitudes for the two phases plateau at a 60/40 distribution at saturating CytC concentrations and not 50/50 may be due to the contribution of flavin semiquinone to the signal at 550 nm, which would be expected to make the amplitude of the first phase appear larger as the semiquinone is produced with the first electron transfer to CytC and would make the second appear smaller as the semiquinone decays with the second electron transfer. Linear fitting of the *k*_obs_ plots produced second order rate constants for the reaction with CytC (*k*_ox_^CytC^) of 10^4^–10^6^ M^–1^ s^–1^, which are comparable to that of Pnao and NicA2, in agreement with these other FAOs also being CytC-using dehydrogenases (Figures 2, 3, Tables 1, 2 and Figure S4). Notably, the range of CytC concentrations used in these experiments (40–80 μM) is not high enough for the pseudo-first order approximation to robustly apply (since the FAO concentration used was 18 μM).^28^ However, obtaining data at CytC concentrations higher than 80 μM was not feasible in these experiments due to the limited CytC yields obtained from bacterial expression and protein purification. A consequence of this is that linear fitting of the *k*_obs_ plots would likely underestimate the true *k*_ox_^CytC^ values since the true slope of the dependence of *k*_obs_ on [CytC] would not fully manifest at sub pseudo-first order reactant concentrations.^29^ Accordingly, the true *k*_ox_^CytC^ values for these reactions may be even higher than those reported in Figure 3 and Tables 1–3, corresponding to even more robust dehydrogenase activities.

**Table 1:** Second Order Rate Constants for Flavin Re-Oxidation by O_2_ and CytC for FAOs from Clade 2

FAO from	*k*_OX_^O_2_^ (M^–1^ s^–1^)	*k*_OX1_^CytC^ (M^–1^ s^–1^)	*k*_OX2_^CytC^ (M^–1^ s^–1^)
*T. dinghuensis*	380 ± 10	2.4 ± 0.1 × 10^5^	3.5 ± 0.3 × 10^4^
*B. anthina*	4.8 ± 0.5 × 10^3^	2.9 ± 0.1 × 10^4^	1.1 ± 0.1 × 10^3^
*E. alkalisoli*	670 ± 50	3.4 ± 0.2 × 10^5^	1.1 ± 0.1 × 10^4^
*B. cepacia* ^a^	1.9 ± 0.1 × 10^3^		
*R. sordida* ^a^	6.1 ± 0.3 × 10^3^		
*Janthinobacterium* sp. PC23-8^a^	1.2 ± 0.1 × 10^4^		
*P. reinekei* ^b^	2.6 ± 0.1 × 10^4^		
*P. putida* OR45a^a^	1.6 ± 0.1 × 10^4^		

a These FAOs lack a signal peptide and genome-associated CytC, so no comparable measurement with CytC could be performed.

b FAO contains a signal peptide but lack a genome-associated CytC in its genome of origin. Thus, no comparable measurement with CytC could be performed.

**Table 2:** Second Order Rate Constants for Flavin Re-Oxidation by O_2_ and CytC for FAOs from Clade 3

FAO from	*k*_OX_^O_2_^ (M^–1^ s^–1^)	*k*_OX1_^CytC^ (M^–1^ s^–1^)	*k*_OX2_^CytC^ (M^–1^ s^–1^)
*Pseudomonas* sp. GL-RE-19	24 ± 1	3.5 ± 0.2 × 10^5^	6.8 ± 0.7 × 10^4^
*K. lipolytica*	1.8 ± 0.1 × 10^3^	1.0 ± 0.1 × 10^6^	1.3 ± 0.1 × 10^5^
*M. nitratireducens*	390 ± 10	4.3 ± 0.3 × 10^5^	3.9 ± 0.3 × 10^4^
*P. taiwanensis*	6.6 ± 0.3 × 10^3^	2.8 ± 0.3 × 10^6^	3.7 ± 0.2 × 10^5^
*P. nitroreducens*	1.9 ± 0.1 × 10^3^	7.2 ± 0.4 × 10^5^	2.1 ± 0.1 × 10^4^
*T. aromatica*	1.6 ± 0.1 × 10^3^	4.7 ± 0.3 × 10^5^	1.1 ± 0.1 × 10^5^
NicA2 (*P. putida* S16)^a^	27 ± 1	1.0 ± 0.2 × 10^6^	2.7 ± 0.2 × 10^5^
Pnao (*P. putida* S16)^b^	600 ± 40	1.4 ± 0.2 × 10^5^	3.2 ± 0.9 × 10^4^
NctB (*Shinella* sp. HZN7)^c^	1.9 ± 0.1 × 10^3^		
*A. digitatis* ^c^	1.6 ± 0.1 × 10^4^		
*Sphingopyxis* sp. YR83^d^	2.7 ± 0.1 × 10^3^		
*Streptomyces* sp. CJ13^c^	5.1 ± 0.1 × 10^4^		
*Synechoccocus* sp. UW140^c^	5.6 ± 0.1 × 10^4^		
*Erythrobacter* sp. 1414^d^	470 ± 10		
*R. tatauoinensis* ^d^	3.1 ± 0.1 × 10^3^		

a From ref 7.

b From ref 10.

c These FAOs lack a signal peptide and genome-associated CytC, so no comparable measurement with CytC could be performed.

d These FAOs contain a signal peptide but lack a genome-associated CytC in their genome of origin. Thus, no comparable measurement with CytC could be performed.

**Table 3:** Second Order Rate Constants for Flavin Re-Oxidation by O_2_ and CytC for FAOs from Clade 1

FAO from	*k*_OX_^O_2_^ (M^–1^ s^–1^)	*k*_OX1_^CytC^ (M^–1^ s^–1^)	*k*_OX2_^CytC^ (M^–1^ s^–1^)
*B. metallica*	320 ± 10	990 ± 140	
*P. aeruginosa*	160 ± 10	290 ± 30	
*B. stagnalis*	590 ± 30	2.7 ± 0.2 × 10^3^	210 ± 20
*P. otitidis* ^a^	4.5 ± 0.1 × 10^3^		

a FAO from contains a signal peptide but no detectable CytC close to the FAO in its genome. No comparable measurement therefore could be performed with CytC.

In contrast, each of these FAOs reacted considerably slower with O_2_ than they did with their associated CytC, with reaction traces occurring in a single phase with *k*_obs_ that varied linearly with O_2_ concentration (Figure 2). The second order rate constants for reaction with O_2_ (*k*_ox_^O_2_^) for the FAOs from , , *Pseudomonas* sp. GL-RE-19 and were comparable to those of NicA2 and Pnao (24–670 M^–1^ s^–1^), and much lower than that typically observed in bona fide flavoprotein oxidases (Figures 2, 3, Tables 1, 2 and Figure S4). This, combined with their rapid reaction with CytC strongly suggests that they are CytC-using dehydrogenases. The putative dehydrogenases from , , and had *k*_ox_^O_2_^ values on the order of 10^3^ M^–1^ s^–1^, which is roughly an order of magnitude larger than that of NicA2 and Pnao and is on the lower end of what is typically observed in flavoprotein oxidases (∼10^4^–10^6^ M^–1^ s^–1^).^31^ However, *k*_ox1_^CytC^ for these enzymes is 290–550 times larger than the corresponding *k*_ox_^O_2_^ value, indicating that CytC is strongly preferred as the oxidant for these enzymes and making it unlikely that they function as oxidases in vivo. Unlike the other putative dehydrogenases, the FAO from reacts relatively rapidly with O_2_ with *k*_ox_^O_2_^ of 4.8 × 10^3^ M^–1^ s^–1^ but also reacts with its genome associated CytC protein with *k*_ox_^CytC^ values comparable to its *k*_ox_^O_2_^ (Table 1 and Figure 3). This observation suggests that FAO may have both oxidase and dehydrogenase activities.

The putative dehydrogenases tested from clade 1 i.e., , and reacted slowly with O_2_, producing *k*_ox_^O_2_^ values (160–590 M^–1^ s^–1^) that were comparable to that of free flavin (*k*_ox_^O_2_^ ∼ 250 M^–1^ s^–1^);^32^ however, they also reacted modestly with their associated CytC proteins (*k*_ox1_^CytC^ values 290–2700 M^–1^ s^–1^) (Table 3, Figures 3 and S4). Notably, the cytochromes associated with the putative dehydrogenases in clade 1 are predicted to be ∼20 kDa, membrane bound diheme-containing c4-type cytochromes instead of the prototypical ∼11 kDa monoheme class I-type CytC proteins associated with the dehydrogenases in clades 2 and 3.^33^ The fact that the c4 type cytochromes are thought to be bound to the inner membrane in vivo^34^ may explain the poor reactivity observed between FAO-CytC pairs in clade 1, as our kinetic analyses were solution-based measurements that fail to mimic the environment near membranes. However, sluggish reactivity with O_2_ suggests that these enzymes do not employ dioxygen as their physiological electron acceptor.

### Putative Oxidases React Efficiently with O_2_

Many FAOs from the dehydrogenase containing clades lacked adjacent CytC genes and/or TAT signal peptides (black branches in Figure 1), leading us to hypothesize that these enzymes function as oxidases. Representative FAOs from these subclades were therefore assessed for their oxidase activity through stopped-flow experiments to measure *k*_ox_^O_2_^. In contrast to their close dehydrogenase relatives, the oxidase reactions all occurred rapidly with completion times ranging from 0.8 to 1.6 s (Figures 4 and S5). The *k*_ox_^O_2_^ values for FAOs from OR45a, *Janthinobacterium* sp. PC23-8, *Streptomyces* sp. CJ13, *Synechoccocus* sp. UW140 and ranged from 10^4^–10^5^ M^–1^ s^–1^, which is comparable to that of bona fide oxidases^31^ (Tables 1, 2 and Figure 3). Notably, these FAOs do not have a genome adjacent CytC gene that can be used for direct comparison. However, the fact that they react quickly with O_2_ and lack an adjacent CytC gene suggests that these FAOs indeed use O_2_ as an oxidant instead of CytC. The putative oxidases from and did not react quite as efficiently with O_2_ and had more modest *k*_ox_^O_2_^ values of 6.1 × 10^3^ and 1.8 × 10^3^ M^–1^ s^–1^, respectively (Table 1 and Figure 3). It has been observed in some FAOs that substrate or product binding in the active site accelerates FADH_2_ oxidation by O_2_, e.g., FADH_2_ oxidation in human monoamine oxidase A (MAO-A) is enhanced ∼125-fold in the presence of kynuramine.^30^ In addition, 6-l-hydroxynicotine oxidase, NctB, from *Shinella* sp. HZN7 in clade 3 has previously been established as an oxidase,^27^ and we observed a ∼27-fold increase in *k*_ox_^O_2_^ for this enzyme when its natural product, 6-hydroxy-*N*-methylmyosmine, is bound to the enzyme (Figure 4d). Similar behavior is also observed with the distantly and independently evolved 6-l-hydroxynicotine oxidase from^35,36^ (6LHNO; see Figures 1a and S5j). It is therefore conceivable that *k*_ox_^O_2_^ would similarly be accelerated for the FAOs from and , and perhaps the other enzymes, when their as-yet unknown substrate/product is bound.

**Figure 4:**
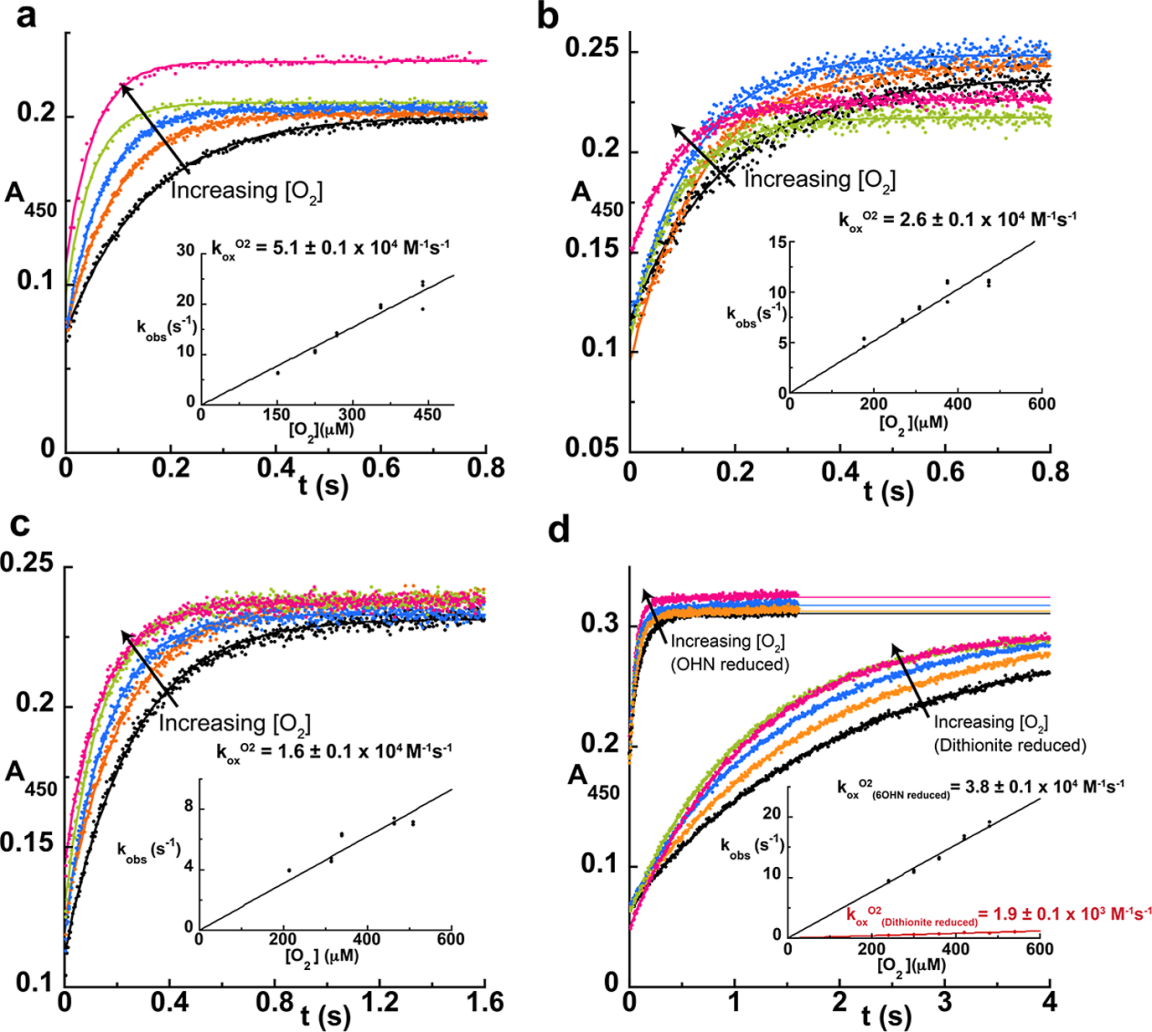
Kinetics of reoxidation for representative putative oxidases by O_2_. Overlay reoxidation reaction traces of flavin hydroquinone for exemplar putative oxidases from (a) *Streptomyces* sp. CJ13, (b) (c) OR45a and (d) *Shinella* sp. HZN7 NctB traces for the reaction with O_2_ monitored at 450 nm. The insets show the dependence of the observed rate constant on oxygen concentration and linear fitting provides the bimolecular rate constant for the reaction with oxygen (*k*_ox_^O_2_^). The product resulting from amine oxidation is known for NctB, unlike the other FAOs, so measurements were conducted in the presence and absence of product to determine if product binding impacts the kinetics of reaction with O_2_. See also Figure S5 for data on additional putative oxidases.

We also characterized the kinetics of reaction with O_2_ for representative FAOs from subclades that contained TAT signal peptides, but did not have a gene for CytC nearby in the genome of origin (see red branches in Figure 1), to assess if these FAOs are periplasm-localized oxidases. Most FAOs in this category tested—, , *Sphingopyxis* sp. YR583, and —had large *k*_ox_^O_2_^ values comparable to that of putative oxidases lacking signal peptides (Figures 3, 4, Tables 1–3 and Figure S5). This suggests that the FAOs containing TAT peptides without adjacent CytC proteins are indeed periplasm-localized oxidases. An exception is the FAO from *Erythrobacter* sp. 1414, which had a *k*_ox_^O_2_^ value comparable to the dehydrogenases (Figures 3 and S5d), suggesting that this periplasm-localized FAO may use an as-yet unknown periplasm-localized oxidant.

### Putative Oxidases React Poorly with CytC

The oxidases from , *Shinella* sp. HZN7 and are closely related to dehydrogenases (Figures 1 and 3). To test whether these enzymes can also reduce CytC, we measured the kinetics of their reaction with the CytC associated with a dehydrogenase from within the same clade. These three putative oxidases reacted poorly with CytC proteins associated with closely related dehydrogenases, with the reactions not reaching completion within the 400 s time period used for data collection. The amplitudes of the reaction traces were also much smaller than those in the reactions between putative dehydrogenases and their genome-adjacent CytC proteins, indicating that FADH_2_ is only partially oxidized when the putative oxidases react with CytC proteins from other organisms (Figure 5 and Table S1). These two observations suggest that the putative oxidases in clades 1–3 are unlikely to act as dehydrogenases in vivo.

**Figure 5:**
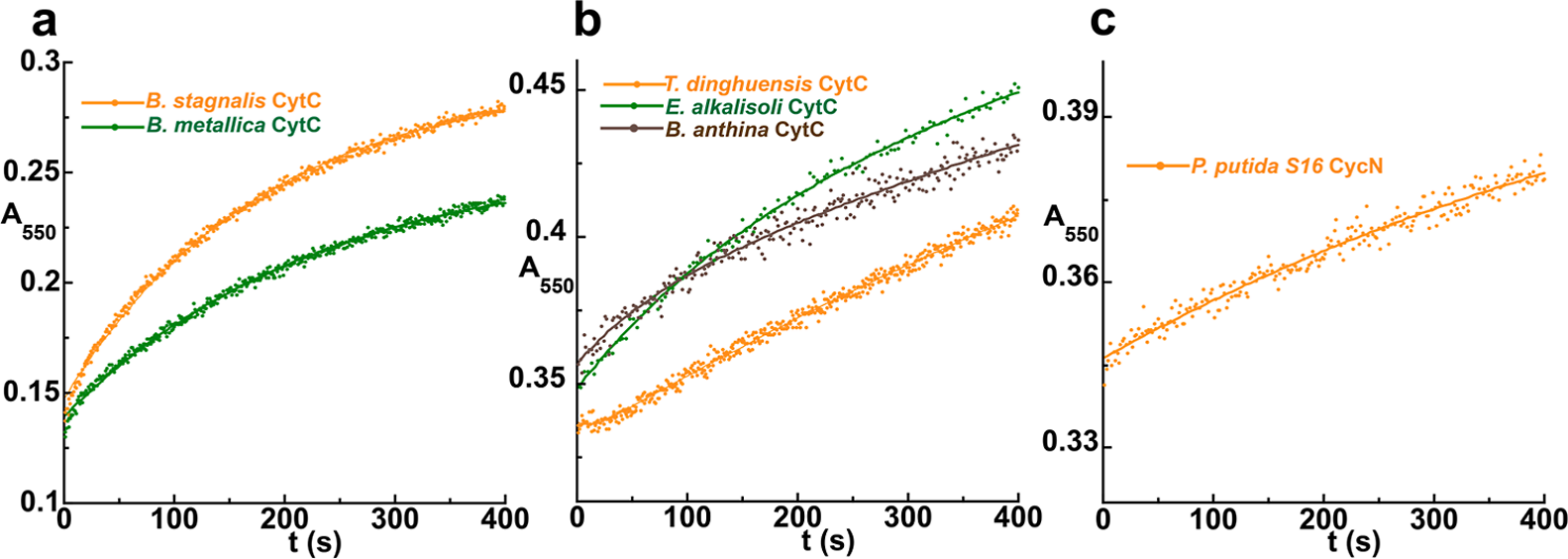
Reaction of FAO-type oxidases with CytC proteins from other organisms within the same clade. (a) FAO (b) FAO (c) NctB from *Shinella* sp. HZN7. Reactions were initiated by mixing 18 μM FAO containing FADH_2_ with 60 μM CytC protein. *k*_obs_ values determined from exponential fitting of the traces are listed in Table S1.

### Dehydrogenases Display Specificity for their Genome-Associated CytC

We next investigated if the newly identified dehydrogenases are specific for the operon-encoded CytC protein from within the same organism, or if the dehydrogenases are promiscuous in their use of CytC as an oxidant. This was done by comparing the reactivity of a given dehydrogenase with CytC from the same organism with the reactivity of that dehydrogenase with CytC proteins derived from other species. Most of the dehydrogenases exhibited slow reactions with CytC proteins from other species, failing to complete within 400 s, thus demonstrating a strong preference for the genome-encoded CytC from the same organism (Figure 6 and Table S1). Most reaction traces fitted to a single exponential function, indicating that the two one electron transfers observed with native FAO-CytC pairs are not detectable when using CytC from another species, presumably due to the low reaction rates. The FAO from is a curious exception to this behavior. This FAO reacted fairly quickly with the CytC proteins from and *Pseudomonas* sp. GL-RE-19, though not quite as fast as with its associated CytC in the genome of (Figure 6g). In comparison, FAO reacted poorly with CytC proteins from and , with reaction traces occurring in one phase. Interestingly, the sequence identities of the CytC proteins from (42.1%), *Pseudomonas* sp. GL-RE-19 (39.8%), (65.7%) and (35.2%) relative to the CytC do not correlate with the relative reactivity of these four noncognate CytC proteins with FAO. AlphaFold models of select FAO-CytC complexes predict that CytC proteins bind to FAOs at the same site predicted and experimentally verified for the NicA2-CycN complex,^9^ suggesting that the FAO-CytC binding interface is conserved among proteins from different organisms. Nonetheless, the structural models show that the residues lining the predicted binding region between FAO and CytC vary considerably between species, potentially explaining the strong preference displayed by dehydrogenases for CytC from the same organism (Figure 7).

**Figure 6:**
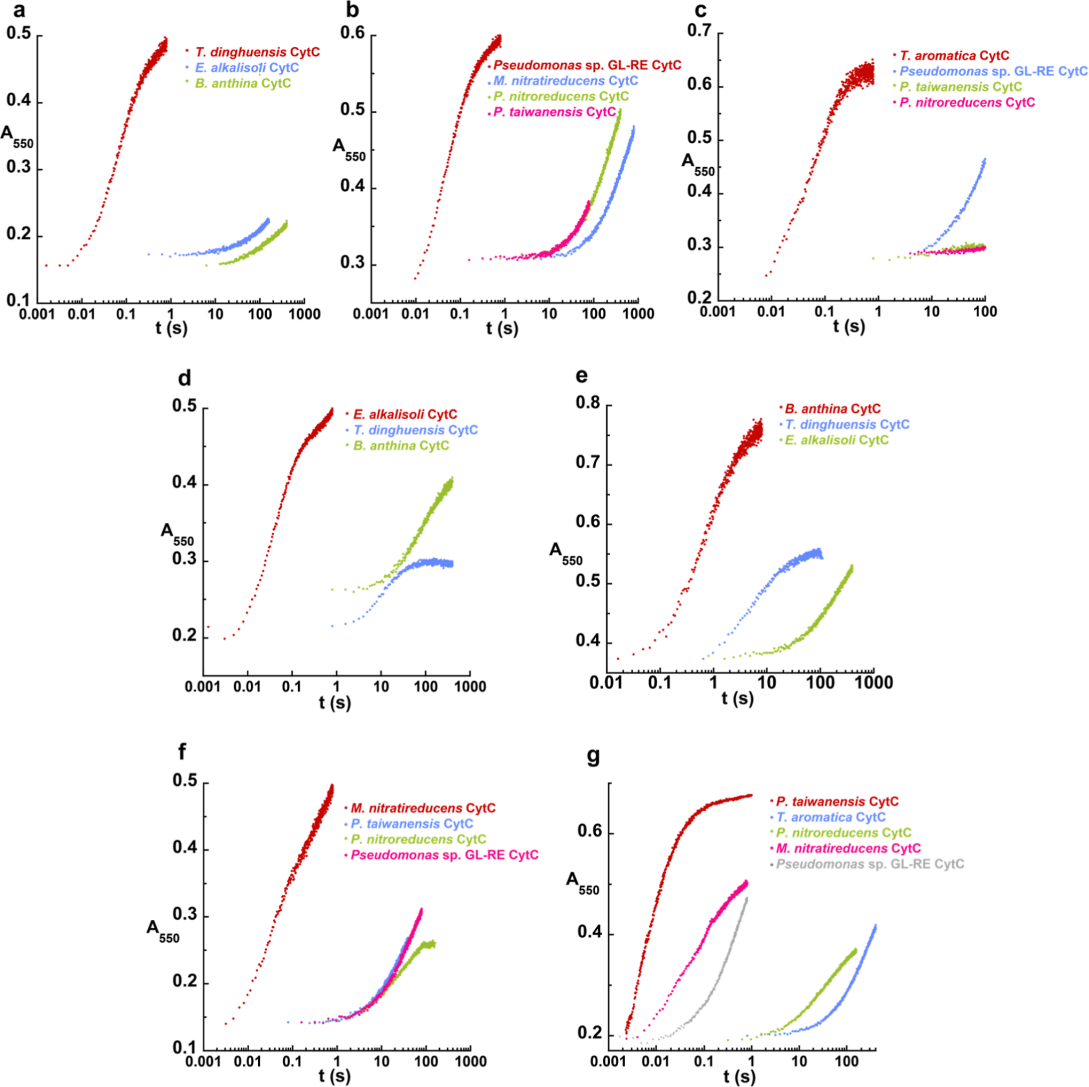
FAO-type dehydrogenases exhibit specificity for their genome-associated CytC. Comparison of FAO-Fl_red_ reoxidation reaction traces by CytC from the same genome (red traces) or CytC from other related species (green, blue, magenta). Data for FAOs from (a) (b) *Pseudomonas* sp. GL-RE 19 (c) (d) (e) (f) and (g) are displayed. The non-native CytC proteins were chosen from species that are within the same clade as the putative target dehydrogenase. All reactions were carried out with 18 μM FAO-Fl_red_ and 60 μM CytC. *k*_obs_ values determined from exponential fitting of the traces are listed in Table S1. Note the logarithmic time scale.

**Figure 7:**
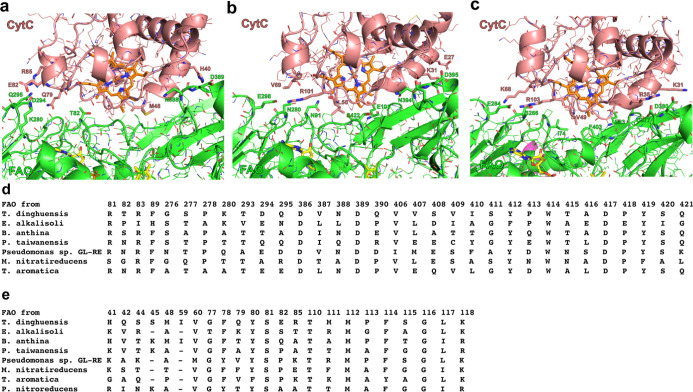
FAO-CytC pairs have variable binding interface residues. The AlphaFold predictions of the FAOs from (a) , (b) *Pseudomonas* sp. GL-RE-19 and (c) in complex with the genome-encoded cytochrome *c* exhibit variable amino acids residues on the FAO-CytC binding interface. (d,e) A list of residues at the binding interface of FAO and CytC, respectively, are shown for characterized dehydrogenase FAO-CytC pairs. Numbers on the top correspond to sequence positions for the proteins from .

## Discussion

By exploring the genetic indicators linked to the discovery of the physiological electron acceptor of NicA2 and Pnao, we have demonstrated that many members of the FAO superfamily are, in fact, CytC-using dehydrogenases. These findings suggest that the utilization of CytC as a physiological electron acceptor by NicA2 and Pnao is not an isolated discovery as previously perceived.^7,10^ Instead, it represents a widely recurring phenomenon within the family. Among the two genetic signposts used here, presence of an adjacent CytC gene appears to be a stronger predictor of dehydrogenase function in bacteria, because FAOs containing only TAT signal peptides without a nearby cytochrome *c* reacted rapidly with O_2_, comparable to that of other well-characterized oxidases in the FAO superfamily. We acknowledge that while these FAOs react quickly with O_2_, other oxidants were not tested and therefore cannot be ruled out. The fact that the dehydrogenases are present in three distinct clades and are interspersed among oxidases indicates that both dehydrogenase and oxidase function has independently evolved several times among the FAO lineages (Figures 1 and 3). However, the use of CytC instead of O_2_ in these organisms may provide a fitness advantage, as it would enable the electrons liberated by FAOs through amine substrate oxidation to be funnelled into the ETC for additional ATP production instead of wastefully reducing O_2_ to H_2_O_2_. The necessity of *cycN* for efficient NicA2 and Pnao activity in S16 confirm that the in vitro measured rates are physiologically meaningful in vivo.^7,10^

Our discoveries reported here raise the possibility that other FAOs from other organisms may also be misinterpreted as oxidases when they are actually dehydrogenases. Bacteria frequently encode genes that function together into operons to facilitate their regulation, which led to our discovery of amine dehydrogenases here; however, eukaryotes do not necessarily utilize similar colocalization in their genomes. Moreover, the sequence and structural features that govern oxidase and dehydrogenase activity in FAOs are poorly understood. This makes attempting to predict these attributes in FAOs from primary sequences alone unreliable and thus requires biochemical or biological characterization of a given eukaryotic FAO to determine if it is an oxidase or dehydrogenase. Notably, MAO, which is anchored to the outer mitochondrial membrane, is the prototype of the FAO superfamily and was first characterized in 1928 as an oxidase involved in neurotransmitter metabolism.^4^ However, it was recently shown that MAO in neurons does not generate H_2_O_2_ upon dopamine oxidation as previously thought, but instead the energy is used to drive the ETC.^37^ The mechanism by which electrons get from MAO to the ETC is currently unknown, but this observation suggests that MAO may actually be a dehydrogenase that uses an oxidant other than O_2_ in vivo.

An open question resulting from this work is the mechanism by which these enzymes modulate the reactivity of their flavin cofactor with O_2_. For most of these FAOs, it is unclear why some enzymes react poorly with O_2_ whereas others react quickly, as the residues proximal to the flavin C4a-N5 locus where the reaction with O_2_ occurs are frequently conserved between the dehydrogenases and oxidases that were functionally characterized in this study (Figure 8), suggesting that the rate of reaction with O_2_ in these enzymes is not modulated by specific amino acid changes near the cofactor. In support of this, we previously identified residues in NicA2 that suppress its reaction with O_2_ through an experimental evolution approach and identified a combination of residues that prevent its flavin from reacting quickly with O_2_, several of which are distant from the flavin C4a-N5 locus.^8^ This indicates that control of O_2_ reactivity in these enzymes is multifaceted and is not exclusively confined to the active site, making it difficult to identify structural features that control the reaction with O_2_ from sequences alone. Complicating things further, our phylogenetic and biochemical data indicate that oxidase activity has independently evolved multiple times in these dehydrogenase containing lineages (Figures 1 and 3) and it appears that some oxidases have higher sequence identity with their related dehydrogenases than with other oxidases. For example, the established oxidase NctB from *Shinella* sp. HZN7 (subclade 3H in Figure 1) is 42% identical to the NicA2-like dehydrogenases (subclade 3G in Figure 1), on average, while its identity to the next most closely related oxidases in subclade 3J only averages 31%. Thus, it is conceivable that each oxidase-containing subclade evolved an independent mechanism of accelerating the reaction with O_2_. However, several mechanistic studies on specific members of the FAO superfamily have identified a lysine residue adjacent to the flavin N5 as playing a key role in enhancing the reaction with O_2_ for bona fide oxidases of this superfamily.^38–41^ This lysine residue (Lys340 in NicA2) is nearly universally conserved among FAO superfamily enzymes and is present in nearly all amine dehydrogenases (including NicA2 and Pnao) and oxidases identified in this study. However, this lysine has been replaced by a glutamate in the FAO from *Pseudomonas* sp. GL-RE-19 (Figure 8b) and a few other FAOs within subclade 3A (Figure 1), and this substitution may help explain why this FAO has the lowest *k*_ox_^O_2_^ value of any enzyme tested.

**Figure 8:**
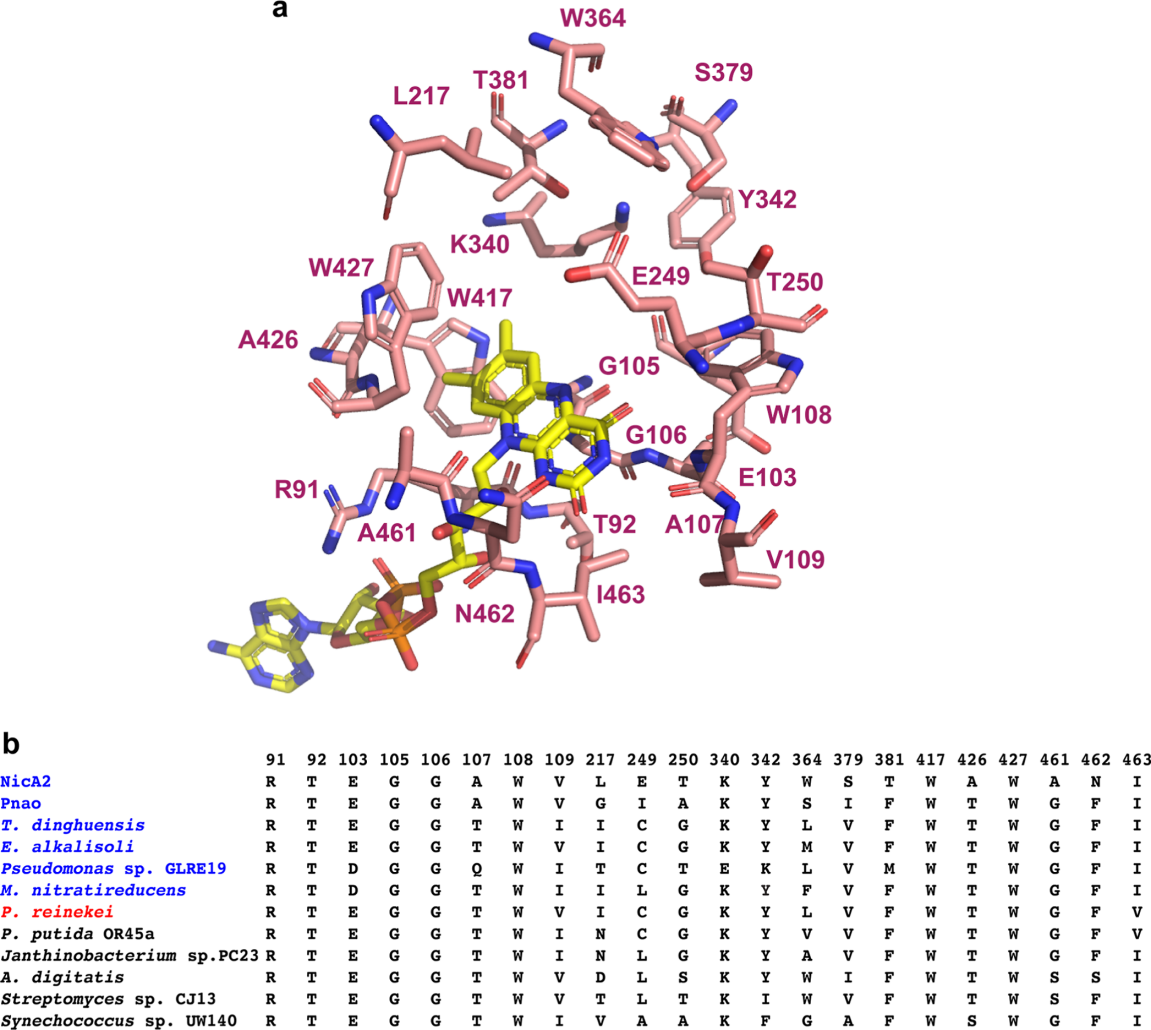
Residue identities for sequence positions close to the flavin C4a-N5. (a) Structure of NicA2 (PDB 7C4A) displaying residues with side chains <10 Å from the flavin N5. (b) Sequence alignment of residues predicted to be <10 Å from the flavin N5 for amine dehydrogenases (colored blue) and amine oxidases (red and black). Only the six dehydrogenases with lowest *k*_ox_^O_2_^ and six oxidases with highest product free *k*_ox_^O_2_^ are shown in this alignment to facilitate comparison. Residue numbering is based on the full length NicA2 sequence.

Most of the flavoprotein amine dehydrogenases we have documented exhibit a high degree of preference for the genome-adjacent CytC within the same operon and react poorly with CytC proteins from other organisms. Several of the sequence positions on the surface of the FAOs and CytC proteins that line the predicted FAO-CytC binding interface^9^ vary considerably across proteins from different species (Figure 7), which could explain the high degree of specificity displayed by dehydrogenases for the CytC protein from the same organism, as the residues on the surface of each FAO-CytC pair may have evolved to be specifically complementary to each other as a means of enabling productive binding between the two proteins. Interestingly, FAO is able to react relatively quickly with the CytC proteins from and *Pseudomonas* sp. GL-RE-19 but not the ones from or , despite the fact that the CytC from is much more closely related to the CytC from (65.7% sequence identity) than the CytC proteins from and *Pseudomonas* sp. GL-RE-19 (42.1% and 39.8% identity to CytC, respectively) (Figure 6g). This may indicate that only specific sites on these CytC proteins play a role in determining whether they react efficiently with the FAO. Conversely, the FAOs from and *Pseudomonas* sp. GL-RE-19 react poorly with the CytC from (Figure 6f,b), suggesting that the specificity conferring sequence positions may be different between the various dehydrogenases. If true, it will make it even more difficult to predict oxidase/dehydrogenase function from the FAO primary sequences alone.

Many other superfamilies of flavoprotein oxidoreductases contain members that are oxidases which react efficiently with O_2_, and dehydrogenases that react poorly with O_2_ and instead utilize a variety of organic molecules as oxidant. Examples are the glucose-methanol-choline (GMC) oxidoreductase,^42^ vanillyl alcohol oxidase (VAO),^43,44^ and α-hydroxy acid oxidoreductase (HAOx)^45^ families of flavoprotein oxidoreductases. It is therefore perhaps not surprising to discover here that the FAO superfamily should be added to that list, and we propose renaming it to the flavoprotein amine oxidoreductase superfamily to more accurately reflect these observations. However, phylogenetic and biochemical studies of the GMC, VAO and HAOx superfamilies have shown that there is usually a single ancestral point of divergence that divides oxidases and dehydrogenases within these superfamilies.^42–45^ Thus, the bacterial FAO containing lineages investigated here are unusual in that there are several ancestral transitions between dehydrogenase and oxidase function; for instance, the FAOs from and *Shinella* sp. HZN7 are oxidases that both appear to have only recently gained such activity since they are nested within clades of dehydrogenases (Figures 1 and 3). These patterns suggest that it is relatively facile for nature to fine-tune the oxidant reactivity of these enzymes depending on the needs of the organism.

## Conclusions

Our investigation has revealed that not all members of the FAO superfamily can be unequivocally categorized as oxidases, as we have demonstrated that many bacterial FAOs are actually cytochrome *c*-using dehydrogenases. The kinetic data reported here indicate that the dual presence of a TAT signal peptide in the FAO and operonic association with a CytC gene are good predictors of dehydrogenase function in bacterial FAOs. We also showed that these newly discovered amine dehydrogenases are largely specific for their operon-encoded CytC, as they generally react poorly with CytC proteins derived from other organisms. Our findings reinforce the value of genomic information in diagnosing enzyme function, and the genetic signposts used here may have utility in identifying CytC-utilizing dehydrogenases in other bacterial flavoprotein oxidoreductase families.

## Supplementary Material



## Notes

Clades 1–3, additional experimental stopped flow data, and a table of reaction rate constants (PDF)

## Abbreviations

FAO: flavoprotein amine oxidoreductase
CytC: cytochrome *c*
FAD: flavin adenine dinucleotide
MAO: monoamine oxidase
FADH_2_: 2-electron reduced FAD hydroquinone
NicA2: nicotine oxidoreductase
Pnao: pseudooxynicotine amine oxidase
ETC: electron transport chain
TAT: twin arginine translocation
FAO-Fl_red_: an FAO containing FADH_2_
*k*_ox_^O_2_^: second order rate constant for the reaction with O_2_
*k*_ox_^CytC^: second order rate constant for the reaction with cytochrome *c*
NctB: 6-l-hydroxynicotine oxidase from *Shinella* sp. HZN7
6LHNO: 6-l-hydroxynicotine oxidase from
