# Supplementary material for: Dehydrogenases in the Flavoprotein Amine Oxidoreductase Superfamily
Source: Biochemistry. 2025 Jun 12;64(13):2834–47. doi: 10.1021/acs.biochem.5c00129 (PMC12224317; doi:10.1021/acs.biochem.5c00129)
Supplement: Supplementary file 1 [file bi5c00129_si_001.pdf]

Supporting Information for

Dehydrogenases in the flavoprotein amine oxidoreductase superfamily

Javeria Akram,[1] Tavishi Budagavi,[1] Zhiyao Zhang,[1] Morgan Fowler,[1] Andrew J. Gaunt,[1] Todd J. Barkman,[2] Frederick Stull[1*]

[1]Department of Chemistry, Western Michigan University, Kalamazoo, MI 49008, USA

[2]Department of Biological Sciences, Western Michigan University, Kalamazoo, MI 49008, USA

* Frederick Stull
Email: frederick.stull@wmich.edu

This PDF file includes:

Figures S1 to S5
Table S1

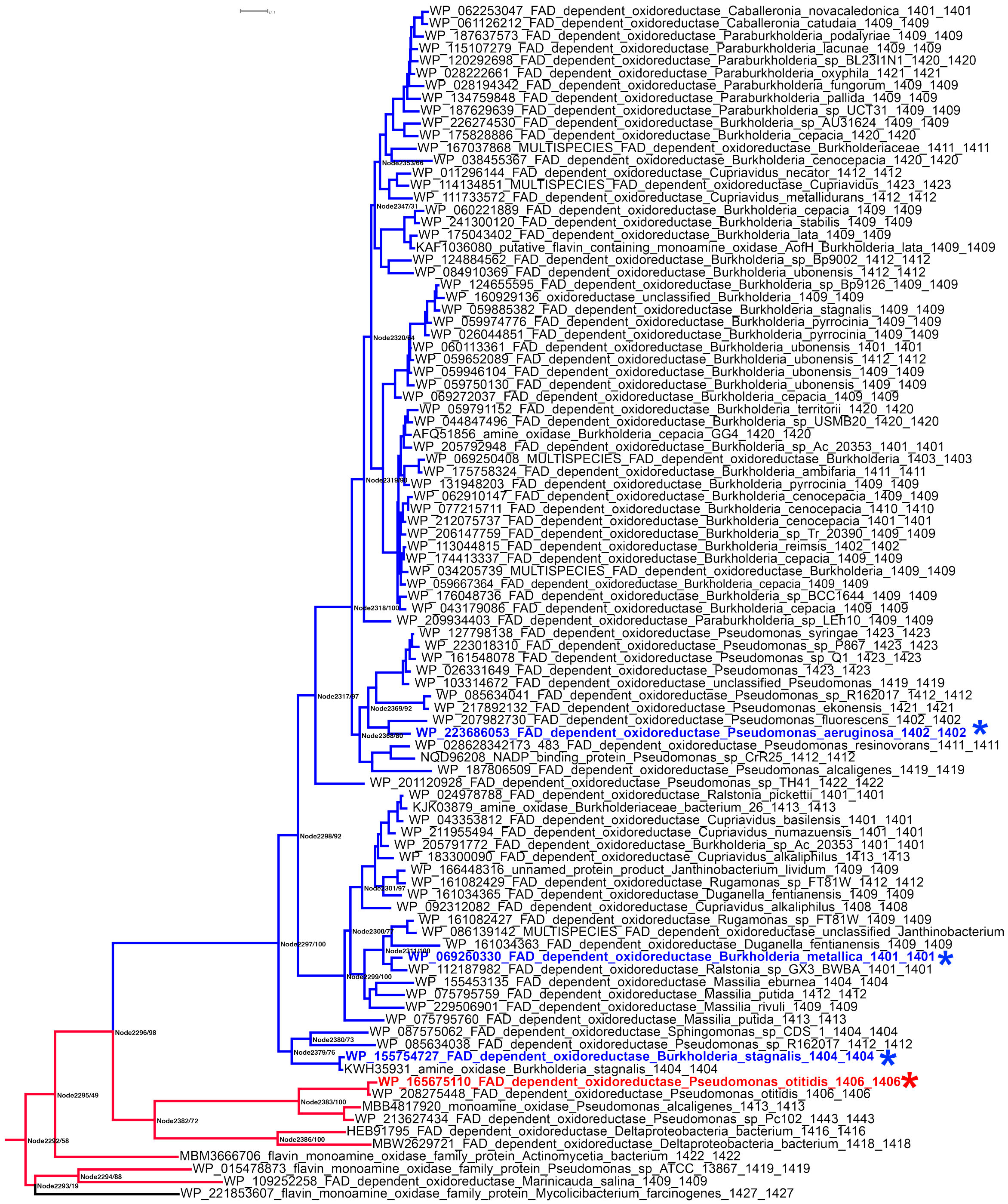

Figure S1. Clade 1. Phylogram displaying all the branches in clade 1. Stars and bolded names indicate the black, blue and red branch FAOs selected for experimental evaluation in this study.

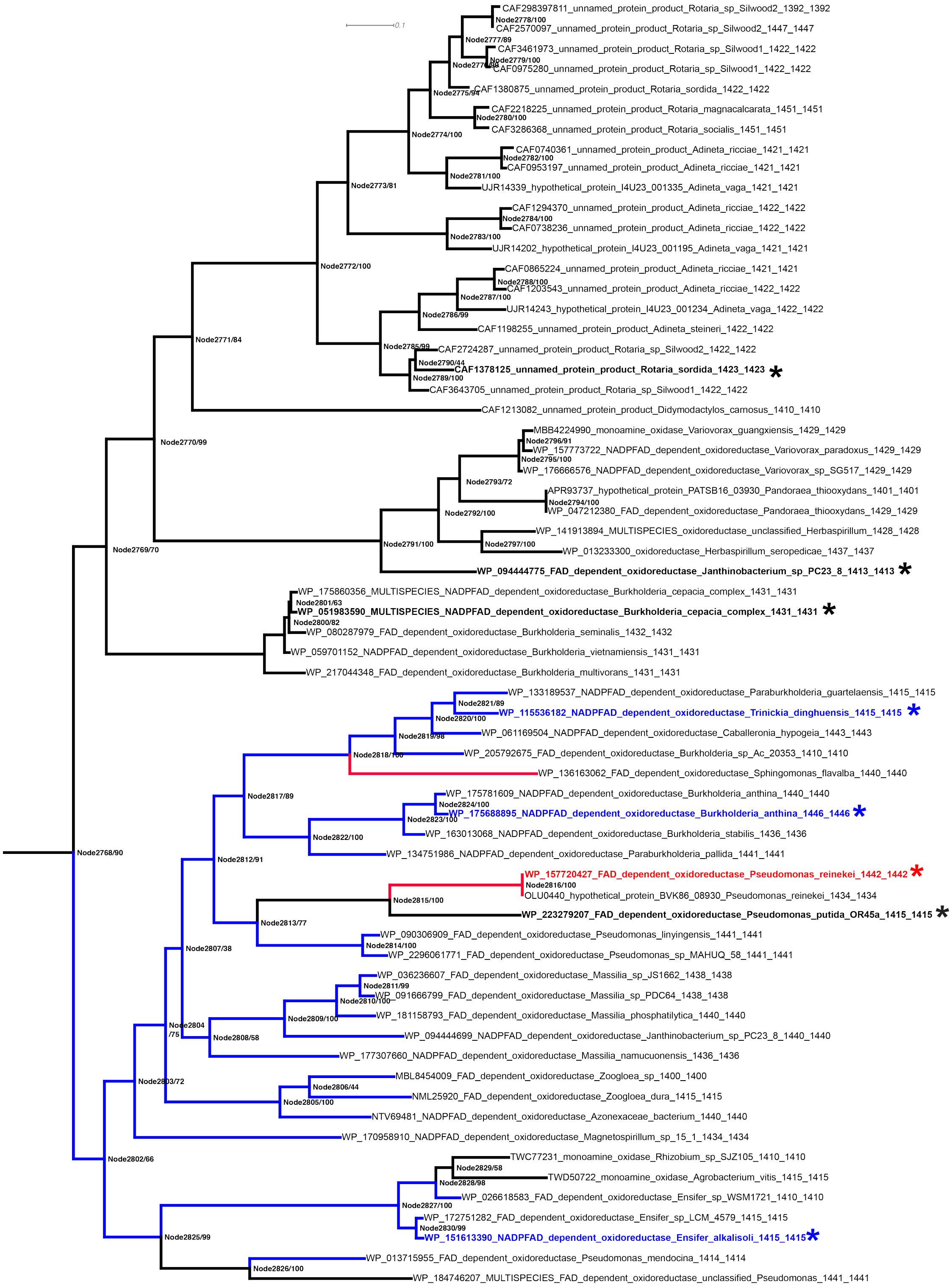

Figure S2. Clade 2. Phylogram displaying all the branches in clade 2. Stars and bolded names indicate the black, blue and red branch FAOs selected for experimental evaluation in this study.

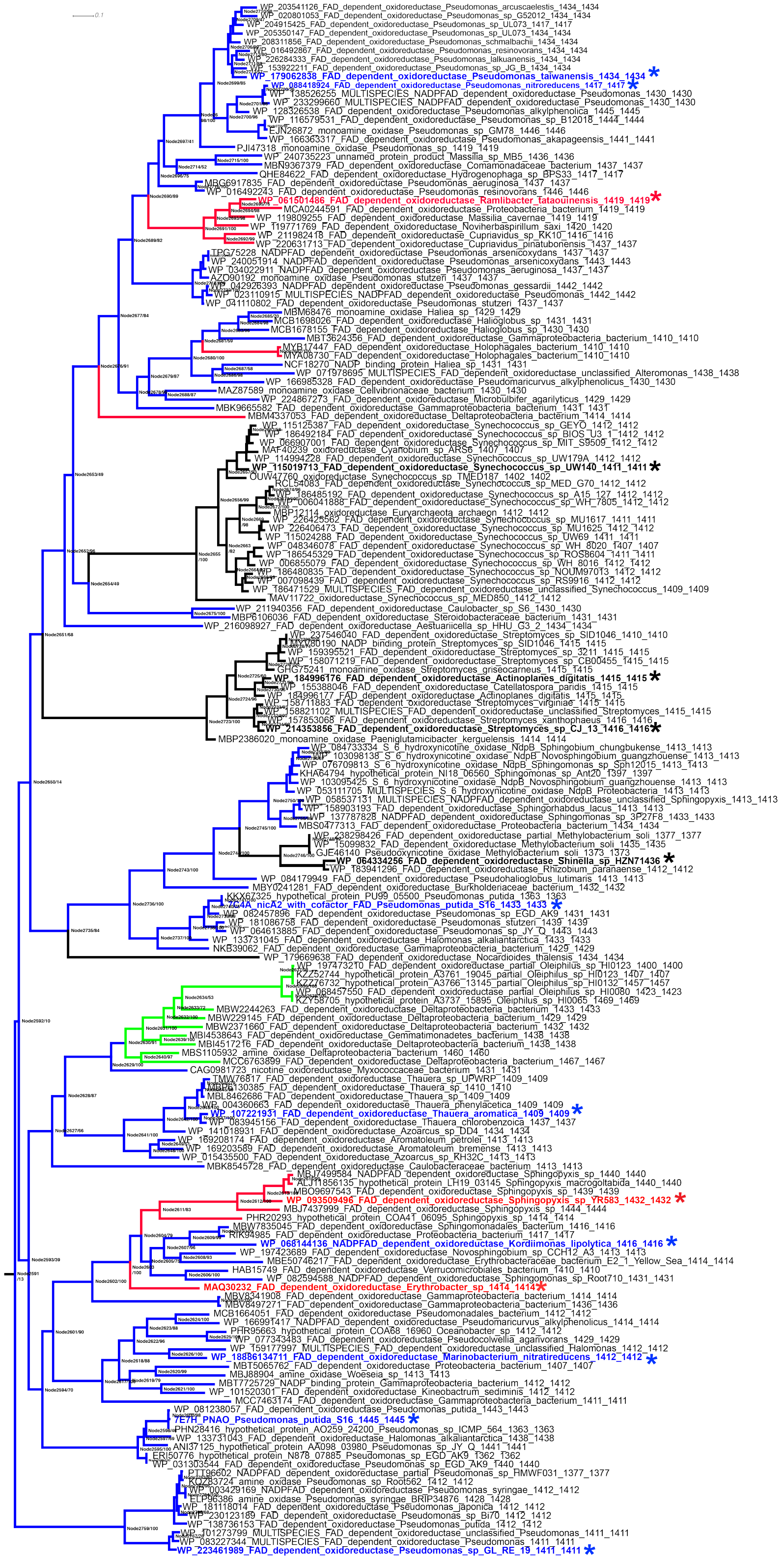

Figure S3. Clade 3. Phylogram displaying all the branches in clade 3. Stars and bolded names indicate the black, blue and red branch FAOs selected for experimental evaluation in this study.

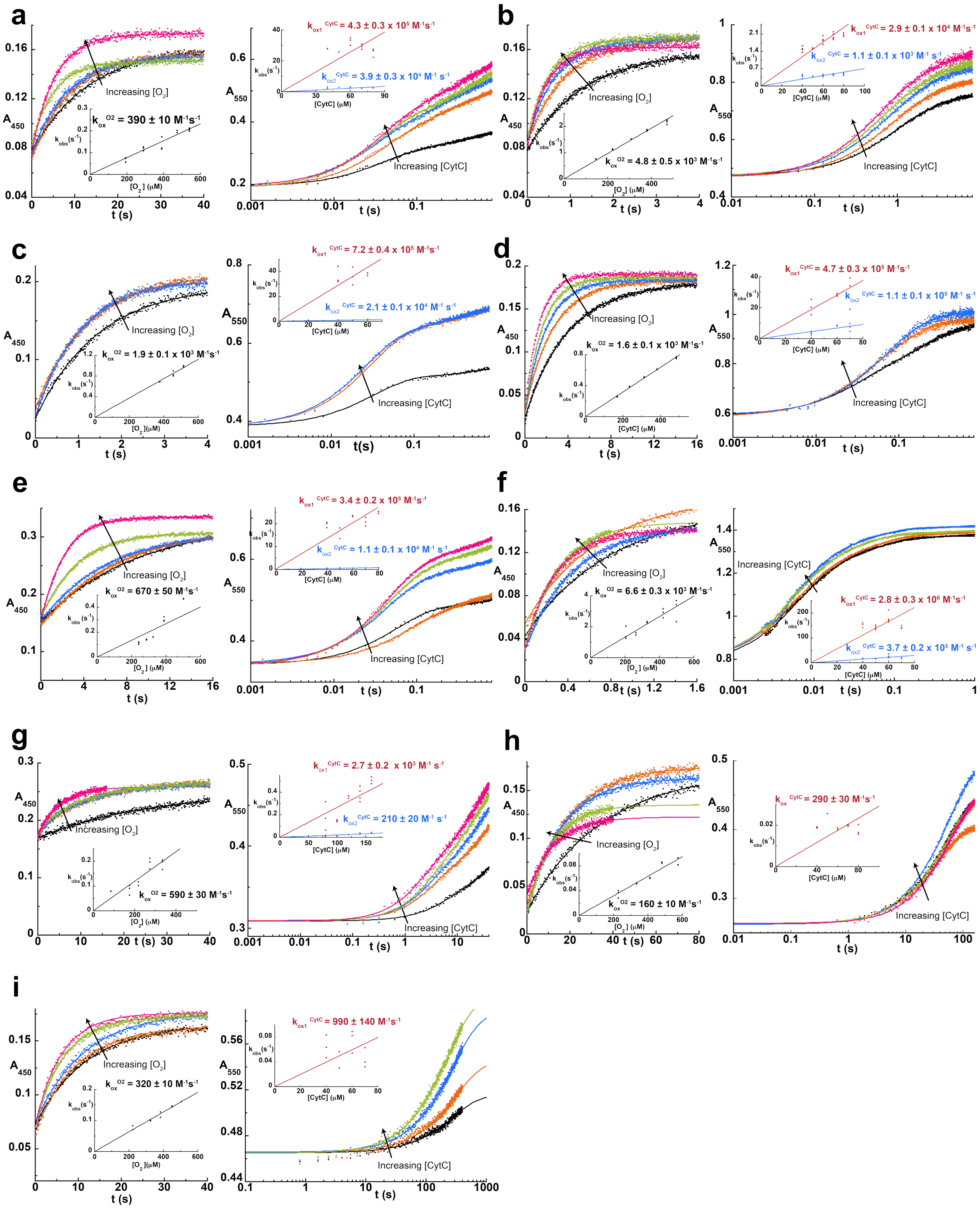

Figure S4. Kinetics of reoxidation for putative dehydrogenases. (a) *M. nitratireducens* (b) *B. anthina* (c) *P. nitroreducens* (d) *T. aromatica* (e) *E. alkalisoli* (f) *P.taiwanensis*, (g) *B. stagnalis* (h) *P. aeruginosa* and (i) *B. metallica* Left, traces for the reaction with O_2 monitored at 450 nm and right, traces for the reaction with associated CytC monitored at 550 nm. Insets show linear

dependance of k_{obs} on O_2 and CytC concentrations which determines the bimolecular rate constant for each reaction. CytC traces were adjusted to begin at the same absorbance value for comparison.

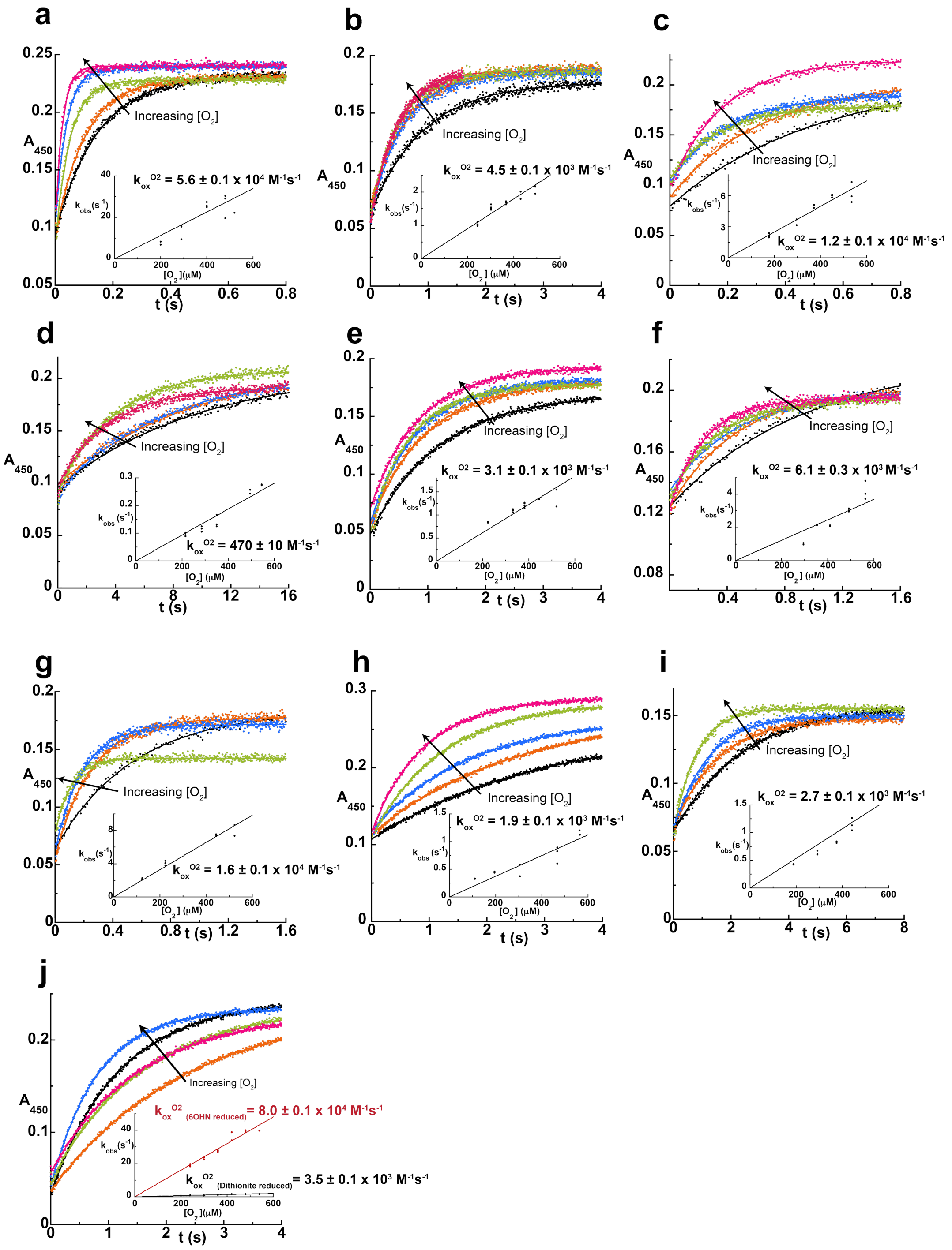

Figure S5. Kinetics of reoxidation by O_2 for putative oxidase ((a) *Synechococcus* sp. UW140 (b) *P. otitidis* (c) *Janthinobacterium* sp. PC23-8 (d) *Erythrobacter* sp. 1414 (e) *R. tatuaensis* (f) *R. sordida* (g) *A. digitatis* (h) *B. cepacia* (i) *Sphingopyxis* sp. YR583 (j) 6LHNO from *P. nicotinovorans* and. Insets show linear dependence of k_{obs} on O_2 concentration, which determines the bimolecular rate constant for each reaction. 6OHN in the inset of panel j denotes 6-hydroxy-L-nicotine.

Table S1. List of observed rate constant (k_{obs}) values from reaction traces for reoxidation of 18 μM reduced FAO by 60 μM CytC.

	FAO from species	CytC species	k_{obs1} (s^{-1})[a]	k_{obs2} (s^{-1})
**Clade 1**	*P. otitidis*[a]	*P. aeruginosa*	0.14	
		B. metallica	0.31	
		B. stagnalis	3.1 x 10^{-2}	
**Clade 3**	*T. dinghuensis*	*T. dinghuensis*	16	2.3
		B. anthina	4.5 x 10^{-3}	
		E. alkalisoli	6.2 x 10^{-3}	
	B. anthina	*B anthina*	2.1	0.51
		T. dinghuensis	0.1	
		E. alkalisoli	4.7 x 10^{-3}	
	E alkalisoli	*E alkalisoli*	23	0.7
		T. dinghuensis	7.8 x 10^{-3}	
		B. anthina	9.7 x 10^{-3}	
	B. cepacia[c]	*T. dinghuensis*	5.7 x 10^{-5}	
		B. anthina	1.6 x 10^{-3}	
		E. alkalisoli	4.7 x 10^{-3}	
**Clade 2**	*T. aromatica*	*T. aromatica*	27.8	8.1
		P. nitroreducens	9.1 x 10^{-4}	
		Pseudomonas sp. GL-RE-19	6.1 x 10^{-3}	
		P. taiwanensis	3.7 x 10^{-3}	
	M. nitratireducens	*M. nitratireducens*	30	2.2
		P. nitroreducens	0.05	
		P. taiwanensis	3.3 x 10^{-3}	
		Pseudomonas sp. GL-RE-19	2.3 x 10^{-2}	
	P. taiwanensis	*P. taiwanensis*	165	24
		T. aromatica	3.1 x 10^{-2}	
		M. nitratireducens	45	3.3
		P. nitroreducens	4.8 x 10^{-2}	
		Pseudomonas sp. GL-RE-19	2.1	
	Pseudomonas sp. GL-RE-19	*Pseudomonas* sp. GL-RE-19	22	9.3
		P. nitroreducens	4.1 x 10^{-3}	
		M. nitratireducens	1.9 x 10^{-3}	
		P. taiwanensis	2.8 x 10^{-3}	
	NdpB *Shinella* sp. HZN7[d]	*P. putida* S16	1.6 x 10^{-3}	

[a]Reactions between FAO and CytC from the same organism fit two a double exponential function and have two k_{obs} values. Reactions between FAO and CytC from another organism fit best to a single exponential function.

[b] *P. otitidis* FAO has a TAT signal peptide but lacks a nearby CytC gene (red branch) and reacts rapidly with O_2.

[c] *B. cepacia* FAO does not have any of the genetic predictors of dehydrogenase function (black branch).

[d] NdpB from *Shinella* HZN7 has been experimentally characterized to be an oxidase[21].

Supplementary References

(1) Qiu, J., Wei, Y., Ma, Y., Wen, R., Wen, Y., and Liu, W. (2014) A Novel (S)-6-Hydroxynicotine Oxidase Gene from *Shinella* sp. Strain HZN7. *Appl Environ Microbiol* (Kelly, R. M., Ed.) *80*, 5552–5560.
